# Lipid Metabolic Disorders and Ovarian Hyperstimulation Syndrome: A Retrospective Analysis

**DOI:** 10.3389/fphys.2020.491892

**Published:** 2020-11-19

**Authors:** Feifei Liu, Qi Jiang, Xuedong Sun, Yuzhen Huang, Zhenzhen Zhang, Ting Han, Yuhua Shi

**Affiliations:** ^1^Center for Reproductive Medicine, Cheeloo College of Medicine, Shandong University, Jinan, China; ^2^Key Laboratory of Reproductive Endocrinology of Ministry of Education, Shandong University, Jinan, China; ^3^Shandong Key Laboratory of Reproductive Medicine, Jinan, China; ^4^Shandong Provincial Clinical Research Center for Reproductive Health, Jinan, China; ^5^National Research Center for Assisted Reproductive Technology and Reproductive Genetics, Shandong University, Jinan, China; ^6^Department of Neurology, Shandong Provincial Hospital, Jinan, China

**Keywords:** lipid metabolism disorders, controlled ovarian hyperstimulation, assisted reproductive technology, ovarian hyperstimulation syndrome, fresh embryo transfer, freeze-all embryo

## Abstract

**Objectives:**

To evaluate the effect of dyslipidemia on the incidence of moderate and severe Ovarian hyperstimulation syndrome (OHSS) in the duration of assisted reproduction technique (ART).

**Methods:**

The study included 233 moderate and severe OHSS patients who received hospitalization after in-vitro fertilization (IVF) or intracytoplasmic sperm injection (ICSI) cycles to avoid severe complications. They were divided into dyslipidemia group and normal lipid metabolism group to evaluate whether dyslipidemia contributes to the development of severe OHSS. Subgroup analysis was set to avoid deviation including the freeze-all group and fresh embryo transfer (ET) group according to whether the eligible women chose fresh embryo transfer immediately after their IVF or ICSI cycles. The main outcome measures included the incidence of moderate OHSS and severe OHSS, total gonadotropin dose, number of oocytes retrieved, age and body mass index (BMI). In the ET groups, the rate of pregnancy is also included for analysis.

**Results:**

In the freeze-all group, lipid metabolism was ultimately identified as the factor affecting the morbidity of severe OHSS and the ones with dyslipidemia were more likely to develop to severe OHSS (*P* < 0.05), while the incidence of severe OHSS among the ET groups had no statistical significance (*P* > 0.05).

**Conclusion:**

The findings of this study suggested that dyslipidemia might contribute to the development of OHSS, especially for those patients who chose the cryopreservation of all embryos. It is essential to consider the risk of OHSS in patients with dyslipidemia although they required cryopreservation of all embryos.

## Introduction

In recent decades, about one in seven couples encounter problems with fertility (European IVF-monitoring programme (EIM) for the European Society of Human Reproduction and Embryology (ESHRE) et al., 2006), and couples increasingly turn to assisted reproductive technology (ART) for help. Complications associated with ART are also becoming more frequent and one of the most serious and common complications during the controlled ovarian hyperstimulation (COH) is ovarian hyperstimulation syndrome (OHSS) (De Mouzon et al., 2012). Ovarian hyperstimulation syndrome (OHSS) is an iatrogenic potentially life-threatening condition resulting from excessive ovarian stimulation. Its reported incidence varies from 1 to 10% in the *in vitro* fertilization (IVF) cycles (Forman et al., 1990; Leya et al., 1991; MacDougall et al., 1992). The main symptoms of OHSS contain abdominal discomfort, nausea, vomiting and sometimes diarrhea, ovarian enlargement, oliguria, fever and shortness of breath (Pellicer et al., 1999; Lee et al., 2008; Herr et al., 2013). In addition, OHSS can also cause serious vascular complications. a rare but devastating consequence of OHSS is thromboembolism, including deep vein thrombosis (DVT), occurring in veins of limbs and even cause cerebral thrombosis, which may lead to permanent injury or death (Chan and Ginsberg, 2006; Chan, 2009). Nowadays, the pathological mechanism of OHSS remains unclear. Overall related literatures, the major pathophysiological mechanism is an increase in capillary permeability, resulting in a shift of fluid from the intravascular space to the extravascular compartments (Delbaere et al., 2005). In previous studies, vascular endothelial growth factor (VEGF) and the cytokine interleukin-6 (IL-6) have been identified as potential mediators in the development of OHSS because of their vasoactive properties, which increase the permeability of capillaries (Motro et al., 1990; Pellicer et al., 1999; Miller et al., 2016). The damage to vascular wall caused by dyslipidemia, characterized by elevated plasma triglyceride (TG) and low-density lipoprotein (LDL) levels, has been proved by researchers worldwide (Battisti et al., 2003; Jenkins et al., 2004; Urbina et al., 2017), and it has become increasingly accepted that high-cholesterol levels can also adversely affect the microvasculature prior to its inducing of proinflammatory (Padró et al., 2018). But it is still unknown that whether dyslipidemia cause the damage of microvasculature in ovary, the correlation between OHSS and lipid metabolism has rarely been reported and the relative effect of lipid metabolism on OHSS therefore demands further examination. In this article, we analyzed 233 cases with moderate and severe OHSS receiving hospitalization after in-vitro fertilization (IVF) or intracytoplasmic sperm injection (ICSI) cycles and compared the degree and results of OHSS occurred in the two groups divided based on the presence or absence of dyslipidemia during ART, aiming to offer some insights on the treatment and prevention of OHSS.

## Materials and Methods

### Study Design and Population

This retrospective study included 233 patients with moderate or severe OHSS (164 moderate and 69 severe) hospitalized after their IVF or ICSI cycles from June 2013 to June 2015 in the Center for Reproductive Medicine, Shandong University. 91/233 women had embryo transfer and the remaining 142/233 patients underwent a “freeze-all” approach. Data was collected after obtaining approval from the medical ethics committee. The eligible women received their cycles of IVF or ICSI because of tubal factors, male factors, or a combination of both. Tubal factors included unilateral or bilateral tubal occlusion, unilateral or bilateral salpingectomy, or peritubal adhesion. Male factors included oligospermia, asthenospermia, or obstructive azoospermia. Women with diagnosis of polycystic ovary syndrome [diagnosed by modified Rotterdam criteria (Rotterdam ESHRE/ASRM-Sponsored PCOS Consensus Workshop Group, 2004; Legro et al., 2014)], adenomyosis, endometriosis, or high risks of OHSS were not included. All patients were divided into 2 groups according to the presence or absence of dyslipidemia. Amongst these cases, 99 cases have dyslipidemia whilst 134 cases were normal in lipid metabolism. The Chinese guidelines on prevention and treatment of dyslipidemia have defined a high concentration of total cholesterol (TC) as ≥240 mg/dL or low-density lipoprotein cholesterol (LDL-C) as ≥160 mg/dL, with or without a low concentration of high-density lipoprotein cholesterol (HDL-C) as ≦40 mg/dL, which were defined as dyslipidemia (Joint Committee for Developing Chinese Guidelines on Prevention and Treatment of Dyslipidemia in Adults, 2016).

### Diagnosis and Classification of Ovarian Hyperstimulation Syndrome

In this study, OHSS was diagnosed by the presence of symptoms, signs, and laboratory findings, traditionally classified into mild, moderate, severe and critical, based on OHSS prevention and treatment guideline of American Society for Reproductive Medicine (Practice Committee of the American Society for Reproductive Medicine, 2016). All recruited patients in our study were moderate, severe and critical OHSS suffering from abdominal pain, nausea and vomiting, ascites, oliguria or anuria, electrolyte disturbances or abnormal liver function. They received hospitalization after their IVF or ICSI cycles with general treatment of bed test, daily assessment of vital signs. Symptomatic treatment, such as circulatory volume correction, electrolyte replacement, anticoagulant therapy, were given according to their clinical symptoms, ultrasound and laboratory examination results.

### Methods

In our center, patients with OHSS risks are given a series of strategies to prevent OHSS. During COH of those patients, gonadotropin-releasing hormone (GnRH) antagonist protocols are preferred which has been proven to reduce the risk of developing OHSS significantly (Weijie et al., 2015; Toftager et al., 2016). However, the standard long-protocol was used in patients with normal ovarian function in our center and antagonist protocol was given in patients with high ovarian response or other OHSS risks, such as PCOS, to prevent the incidence of OHSS. In our study, all patients received a standardized long-term protocol in mid-luteal phase for ovarian stimulation with Gonadotropin-releasing hormone agonist (GnRHa) to down-regulate the functions of the pituitary gland on Day 21 of the previous menstrual cycle. After down-regulation of the pituitary gland, gonadotropin was initiated with a starting dose of recombinant FSH ranging from 75 to 225 IU, and the dose of gonadotropin was modulated according to the patient’s ovarian response. Ovarian response was monitored by serial *trans-*vaginal ultrasound and sex hormones assay were detected as well, which were important for the adjustment of the dose of gonadotropin and the timing for triggering of the final oocyte maturation during ovarian stimulation. Urinary human chorionic gonadotropin (hCG) at a dose of 4000 to 8000IU was administered to induced oocyte maturation when at least two dominant follicles reached 18–20 mm in diameter and oocyte retrieval was performed 36–48 h later. The choice of fresh embryo transfer or freeze-all embryo for patients is determined by ultrasonography and serum estradiol (E_2_) level 2 days after oocyte retrieval. If number of oocytes retrieved ≥15, the serum E_2_ ≥ 5000 pg/ml on hCG trigger day or the serum E_2_ ≥ 1500 pg/ml on the second day after oocyte retrieval, or OHSS manifestations such as nausea, vomiting, abdominal pain and distension appeared, patients would receive a “freeze-all” approach instead of fresh embryo transfer to prevent late OHSS. The positive result of β-hCG (β-hCG ≥ 10IU/ml) in blood showed the biochemical pregnancy 14 days after transplantation, and clinical pregnancy was defined as the presence of a gestational sac in the uterine cavity at 35 days after embryo transfer, as detected through ultrasonography. According to whether or not patients received fresh embryo transfer, we set subgroups of freeze-all group and fresh embryo transfer group for analysis.

### Statistical Analysis

Data analysis was performed using the Statistical Package for Social Science (SPSS) statistics 20. Continuous variables of clinical characteristics were displayed as mean ± standard deviation ($\bar{x}\pm s$) and compared between the dyslipidemia group and the normal lipid metabolism group by *t*-test. Qualitative variables were presented as frequency and percentage and compared between these two groups with different types of lipid metabolism by chi-square test. All values of *P* < 0.05 for two-side tests were considered statistically significant. Among freeze-all groups, to avoid the bias of other characters, such as the total gonadotropin dose and BMI, logistic regression was used to assess the relationships between variables and incidence of severe OHSS. Only variables with *P* < 0.05 were considered to be related to the morbidity of severe OHSS.

## Results

### Comparison of Freeze-All Group and Fresh Embryo Transfer (ET) Group

In the ET group, 59 cases represented moderate OHSS (64.8%) and 32 cases developed severe OHSS (35.2%), while there were 105 cases of moderate OHSS (73.9%) and 37 cases of severe OHSS (26.1%) in the freeze-all group (Table 1). It is showed that the incidence of severe OHSS in the ET group was higher than that in the freeze-all group, but the difference between 2 groups had no significance (35.2% vs. 26.1%, *P* = 0.137). Compared with ET group, the number of oocytes retrieved is significantly higher in the freeze-all group (24.38 ± 9.61 vs. 14.43 ± 5.01, *P* = 0.000), while the age, total gonadotropin dose and BMI between 2 groups had no statistical significance (*P* > 0.05).

**TABLE 1:** Comparison of freeze-all group and fresh embryo transfer group.

Variables	Freeze-all group	Fresh embryo transfer group	*P*-value
Number of cases/N	142	91	0.137
Moderate OHSS	105/142(73.9%)	59/91(64.8%)	
Severe OHSS	37/142(26.1%)	32/91(35.2%)	
Age	29.04 ± 3.48	30.41 ± 4.17	0.055
Total gonadotropin dose (IU)	1724.20 ± 640.32	1751.84 ± 670.16	0.260
Number of oocytes retrieved	24.38 ± 9.61	14.43 ± 5.01	0.000
BMI (kg/m^2^)	23.54 ± 3.80	22.57 ± 3.90	0.944

*OHSS, ovarian hyperstimulation syndrome; BMI, body mass index.*

### Comparison of Freeze-All Groups With Normal Lipid Metabolism or Dyslipidemia

Among the cases who chose freeze-all embryo in this study (Table 2), there were 66 cases of moderate OHSS (80.5%) and 16 cases of severe OHSS (19.5%) in the group of normal lipid metabolism. However, in the dyslipidemia group, there were 39 cases of moderate OHSS (65.0%) and 21 cases of severe OHSS (35.0%). Severe OHSS in the dyslipidemia group was significantly higher than that of the other group (35.0% vs. 19.5%, *P* = 0.038), and the total gonadotropin dose as well as BMI of patients with normal lipid metabolism were markedly lower than those patients in the dyslipidemia group (total gonadotropin, 1603.49 ± 519.49 vs. 1889.17 ± 749.28, *P* = 0.013; BMI,22.55 ± 3.24 vs.24.87 ± 4.15, *P* = 0.000). The logistic analysis (Table 3) of the freeze-all groups showed that after adjusting BMI and total gonadotropin dose, the risk of severe OHSS increased in patients with dyslipidemia compared with patients with normal lipid metabolism (OR:2.22, 95%CI:1.037–4.756).

**TABLE 2:** Comparison of freeze-all groups with normal lipid metabolism or dyslipidemia.

Variables	Freeze-all group with normal lipid metabolism	Freeze-all group with dyslipidemia	*P*-value
Number of cases/N	82	60	0.038
Moderate OHSS	66/82(80.5%)	39/60(65.0%)	
Severe OHSS	16/82(19.5%)	21/60(35.0%)	
Age	28.96 ± 3.68	29.15 ± 3.23	0.754
Total gonadotropin dose (IU)	1603.49 ± 519.49	1889.17 ± 749.28	0.013
Number of oocytes retrieved	24.5 ± 9.58	24.0 ± 9.80	0.678
BMI (kg/m^2^)	22.55 ± 3.24	24.87 ± 4.15	0.000

*OHSS, ovarian hyperstimulation syndrome; BMI, body mass index.*

**TABLE 3:** Logistic repression of severe OHSS risk factors in freeze-all groups.

Variables	B	SE	Wald	OR	95% CI	*P*-value
Lipid metabolism	0.798	0.388	4.220	2.22		0.04
Normal lipid metabolism				1.00	Ref	
Dyslipidemia				2.22	1.037–4.756	
BMI (kg/m^2^)	0.046	0.050	0.862	1.05	0.950–1.154	0.353
Total gonadotropin dose (IU)	0.000	0.000	0.304	1.00	1.000–1.001	0.928

### Comparison of Fresh Embryo Transfer (ET) Groups With Normal Lipid Metabolism or Dyslipidemia

Among the patients with fresh embryos transfer (Table 4), there were 35 cases of moderate OHSS (67.3%) and 17 cases of severe OHSS (32.7%) in the normal lipid metabolism group, whilst there were 24 cases of moderate OHSS (61.5%) and 15 cases of severe OHSS (38.5%) in the dyslipidemia group. The incidence of severe OHSS in dyslipidemia group was higher than that of the other group, but had no statistical significance (38.5% vs. 32.7%, *P* = 0.568). In addition, the age, total gonadotropin dose, number of oocytes retrieved and BMI between these two groups had no significance (*P* > 0.05). Compared with freeze-all groups, pregnancy is also a OHSS risk factor for analysis in ET groups. Among the ET groups, the rate of pregnancy is significantly higher in the normal lipid metabolism group than in the dyslipidemia group (82.7% vs. 74.4%, *P* = 0.000).

**TABLE 4:** Comparison of fresh embryo transfer groups with normal lipid metabolism or dyslipidemia.

Variables	Fresh embryo transfer group with normal lipid metabolism	Fresh embryo transfer group with dyslipidemia	*P*-value
Number of cases/N	52	39	0.568
Moderate OHSS	35/52(67.3%)	24/39(61.5%)	
Severe OHSS	17/52(32.7%)	15/39(38.5%)	
Age	30.25 ± 3.85	30.62 ± 4.60	0.681
Total gonadotropin dose (IU)	1714.87 ± 667.87	1801.10 ± 678.76	0.547
Number of oocytes retrieved	14.31 ± 5.14	14.58 ± 4.89	0.801
BMI (kg/m^2^)	21.96 ± 3.95	23.38 ± 3.74	0.087
Pregnancy	43/52(82.7%)	29/39(74.4%)	0.000

*OHSS, ovarian hyperstimulation syndrome; BMI, body mass index.*

## Discussion

This study assessed the effect of lipid metabolism on the incidence of severe OHSS with 233 participants who received hospitalization after their IVF or ICSI cycles. Among the infertile women in freeze-all group, our study showed that patients with dyslipidemia were more likely to develop severe OHSS. However, the difference of severe OHSS incidence between ET groups was not significant.

According to previous literature, the freeze-all policy is commonly applied for prevention of OHSS. Compared with ET group, freeze-all group had more retrieved oocytes (24.38 ± 9.61 vs. 14.43 ± 5.01, *P* = 0.000) so that we compared characteristics and OHSS incidence within group, respectively, although the incidence of severe OHSS between those 2 groups had no significance in our study. The higher the number of retrieved oocytes, the higher the risk of OHSS. In our center, patients with high number of oocytes retrieved (≥15 generally as mentioned above) would receive freeze-all approach to prevent OHSS. We also found that patients with dyslipidemia had higher BMI and total gonadotropin dose compared with the ones with normal lipid metabolism in the freeze-all groups. BMI and gonadotropin dose have been considered to be the factors to affect the development of OHSS (Practice Committee of the American Society for Reproductive Medicine, 2016). During the ovarian stimulation, doctors will adjust the dose of gonadotropin according to patients’ BMI. Generally speaking, patients with higher BMI need more dose of gonadotropin to promote follicle growth. In Table 2, logistic regression among patients who received freeze-all embryo showed that abnormal lipid metabolism might increase the risk of severe OHSS after adjusting the effect of BMI and total gonadotropin dose, although the two factors mentioned above had statistical significance between the dyslipidemia group and the normal lipid metabolism group.

Pregnancy is a main risk to develop OHSS. After pregnancy, the increase of endogenous HCG, which can stimulate granulosa-lutein cells to produce VEGF and VEGF recepter-2 messenger RNAs to enhance permeability of capillaries, aggravate and prolong the course of OHSS (Namavar et al., 2018). Referring to the comparison of freeze-all groups, the incidence of severe OHSS in patients with dyslipidemia was expected to be significantly higher than that in patients with normal lipid metabolism in ET groups. Among ET groups, the rate of pregnancy is significantly higher in the normal lipid metabolism group than in the dyslipidemia group. Therefore, the incidence of severe OHSS between the two groups with different status of lipid metabolism might be affected, especially when the possibility of pregnancy couldn’t be eliminated in this study. In addition, this research with small sample size in ET groups negated the difference of severe OHSS incidence. Even those were inadequate to explain the effect of lipid metabolism on OHSS development, a larger simple size is needed.

Hypovolemic shock, Liver or renal dysfunction and respiratory distress are severe complications of OHSS (Delbaere et al., 2005; Lee et al., 2008; Rizk and Ebrary, 2011). Recent studies have shown that the offspring of patents with OHSS are less intelligent than the ones of pregnant without OHSS (Xu et al., 2017). Because of the serious consequences, it is of critical importance to pay attention to prevention rather than clinical treatment of OHSS. According to the previous data in our center, the incidence of OHSS is about 1.5–2%. In addition to the factors mentioned above, young age, PCOS, previous OHSS, high level of anti-Müllerian hormone (AMH), elevated serum estradiol (E_2_) are also considered to be risk factors for the development of OHSS (Haning et al., 1983; Forman et al., 1990; MacDougall et al., 1992; Mizunuma et al., 1992; Delvigne et al., 1993; Mordel and Schenker, 1993; Buyalos and Lee, 1996; Whelan and Vlahos, 2000). Using low gonadotropin dose (75IU) in the start of COH, reducing the dose of trigger, changing the trigger time and freeze-all approach are commonly used in our center for those people with high risks of OHSS except GnRH antagonist protocol, and all of those strategies had been proved by researches (Nargund et al., 2007; Howles et al., 2010; Chen et al., 2016; Nelson, 2017; Shi et al., 2018; Zhang et al., 2018). Compared with the fresh embryo transfer, the rates of implantation and clinical pregnancy in freeze-all group were significantly higher (Zhang et al., 2018). Based on those study, patients with dyslipidemia can choose freeze-all embryo, which can shorten the course of early OHSS and prevent late OHSS.

The main strength of the study is that we demonstrated for the first time that dyslipidemia might be a risk factor for developing OHSS among patients without fresh embryo transfers after COH. This has not been confirmed before. Furthermore, all cases in this study received uniform infertility treatment (i.e., the same long-term protocol in midluteal phase for ovarian stimulation, standardized procedures of oocyte retrieval and fertilization, embryo selection, embryo transfer and luteal phase support). Concerned on the influence of fresh embryo transfer versus freeze-all strategy on the incidence of OHSS, we set subgroup analysis to avoid deviation. There are also limitations. The sample size was small, especially the ET group, and there was bias due to single-center analysis. On account of variable risk factors for the development of OHSS, our study mainly focuses on the lipid metabolism without exclusion of other factors such as age, BMI, pregnancy. Although the present study shows evidence favoring the effect of lipid metabolism on the incidence of OHSS among the patients who received freeze-all embryos, the evidence was not enough to establish a definitive causal link between them. In another words, our study try to provides a new perspective for predicting the incidence of OHSS. In addition, the limitations of the retrospective study make it essential to take further large-sample and multicenter clinical investigations to provide strong evidence regarding this aspect.

## Conclusion

Our study suggests that patients with dyslipidemia who received the cryopreservation of all embryos after COH have a higher chance to develop severe OHSS compared with ones with normal lipid metabolism. It is essential to pay attention to the risk of incidence of OHSS in patients with dyslipidemia undergoing ART, especially those women not requiring fresh embryo transfers.

## Data Availability Statement

The datasets generated for this study are available on request to the corresponding authors.

## Ethics Statement

The studies involving human participants were reviewed and approved by Institutional Review Board of Reproductive Medicine Center for Reproductive Medicine, Shandong University. The patients/participants provided their written informed consent to participate in this study.

## Author Contributions

FL conceived and designed the study. FL and QJ analyzed the data. FL, QJ, and XS wrote the manuscript. YH and ZZ took part in the clinical data collection and supervised the entire work. FL, QJ, XS, YH, ZZ, TH, and YS provided final approval for the version to be published. All authors contributed to the article and approved the submitted version.

## Conflict of Interest

The authors declare that the research was conducted in the absence of any commercial or financial relationships that could be construed as a potential conflict of interest.
